# From expectations to experiences: a systematic review of patient and public perspectives on robotic surgery

**DOI:** 10.1007/s11701-025-02649-y

**Published:** 2025-08-14

**Authors:** B. Jauniaux, A. Anand, R. Abbas, D. P. Harji

**Affiliations:** 1 Department of Colorectal Surgery, Manchester University NHS Foundation Trust, Manchester, UK; 2 University of Manchester, Manchester, UK; 3 Robotics and Digital Surgery Initiative, Royal College of Surgeons of England, London, England; 4 Clinical Trials Research Unit, Leeds Institute of Clinical Trials Research, University of Leeds, Leeds, UK

**Keywords:** Robotic surgery, Patient satisfaction, Patient expectations, Patient experiences, Patient perception, Shared decision-making

## Abstract

The adoption of robotic-assisted surgery (RAS) provides opportunities for improved clinical and patient-reported outcomes. However, there are patient and public misconceptions regarding robotic surgery, its associated risks, and surgeon involvement. It is the surgical community’s responsibility to empower and educate patients regarding emerging technologies to improve understanding and shared decision-making. This systematic review aimed to summarize the current understanding, perceptions, expectations, preferences, and experiences of patients and the public with RAS as identified in all primary quantitative, qualitative, and mixed-methods studies and across all surgical specialities. Using the PRISMA 2020 guideline, a mixed-methods systematic review was undertaken with searches conducted in Medline, Embase, and Cochrane Library databases from conception to November 2024. Each study was evaluated regarding its design and methodology. Data quality was reviewed in accordance with the Critical Appraisal Skills Programme Checklist for quantitative and qualitative data, respectively. A narrative synthesis was undertaken using the principles of content analysis to identify emerging themes. Of the 7889 references identified, twelve quantitative, four qualitative, and two mixed-methods studies were included, assessing 2789 patients and 1244 members of the public. All studies were retrospective and assessed cohorts across five specialities, including gynecology, colorectal, urology, orthopedic, and cardiothoracic surgery. Six themes were identified: factual knowledge, awareness, preference, perception and expectations, experiences, and willingness to pay. Patients and the public hold diverse views on robotic-assisted surgery, influenced by multiple factors and various misconceptions. Structured pre-operative education programs generally lead to high satisfaction rates, along with increased knowledge and awareness.

**Supplementary Information:**

The online version contains supplementary material available at 10.1007/s11701-025-02649-y.

## Introduction

Robotic-assisted surgery (RAS) has transformed the field of surgery by providing numerous benefits, including improved precision, reduced post-operative pain, and improved recovery times [1, 2]. In the last two decades, RAS has been adopted across various surgical specialities. RAS has the potential to revolutionize surgical care, but there are significant geographical inequalities in access to this technology globally [3]. The current high cost of robotic platforms limits their availability to well-funded hospitals in urban areas, leaving patients in rural or low-income regions with restricted access [4]. Public awareness of robotic surgery is limited, contributing to patient misconceptions and hesitancy [5, 6]. An essential way to tackle inequalities in access to RAS is through a drive to gather evidence exploring its adoption, benefits, and current limitations, along with innovative projects to increase surgeon and public understanding of the advantages of RAS [6].

Despite growing evidence supporting its clinical advantages, the patient’s perspective encompassing their expectations, attitudes, and experiences remains underexplored [5]. Gaps persist in capturing and addressing the lived experiences and concerns of patients. Studies have suggested that patients perceive robotic surgery as technologically superior; however, there are misconceptions about the actual procedures, the risks, and the surgeon involvement [5, 7, 8]. Moreover, patient perspectives of robotic surgery are undoubtedly influenced by factors, such as age, cultural beliefs, communication with healthcare providers, and exposure to educational materials about technology and alternative surgical approaches. Understanding these psychosocial dimensions is essential to improve patient-centered care and shared decision-making. This, in turn, may reduce misconceptions and align the innovative advancements and adoption of RAS with the holistic needs of patients.

This systematic review aimed to summarize the current understanding, perceptions, expectations, preferences, and experiences of patients and the public regarding RAS, in any setting, as identified in all primary quantitative, qualitative, and mixed-methods studies across all surgical specialities.

## Materials and methods

This mixed-methods systematic review was conducted based on guidance from the Joanna Briggs Institute [9] and is reported in keeping with the Preferred Reporting Items for Systematic Reviews and Meta-Analyses (PRISMA) guidelines and the PRISMA checklist [10, 11]. The review was conducted according to a pre-specified protocol and registered with PROSPERO (CRD42024611837).

### Search strategy and eligibility criteria

A systematic search was conducted in Medline and Embase (via OVID) and the Cochrane Library to identify studies published without time limitation until November 2024. The following medical subject headings (MeSH) terms were used: ‘Robotic Surgical Procedures’, ‘Specialties, Surgical’, ‘Surgical Procedures, Operative’, ‘Patient Satisfaction’ and ‘*Attitudes to Health’. The full search strategy is shown in the Online Resources (Online Resource 1). To ensure all data were captured and to identify any missed articles, citations and reference lists of selected studies were reviewed, and appropriate articles were screened for inclusion.

Following the population, intervention, comparison, outcomes, and study design (PICOS) framework, we established the criteria for study eligibility: *(1)* Studies of at least ten adult patients undergoing robotic-assisted surgery in any surgical speciality, or patients and members of the public interviewed about robotic-assisted surgery in any setting, *(2)* All qualitative and quantitative study designs, *(3)* Studies examining patient satisfaction, experiences or expectations, and attitudes toward the concept of robotic-assisted surgery.

Exclusion criteria were: *(a)* Studies reporting on quality of life or patient-reported outcome measures. *(b)* Studies reporting on patient satisfaction, experiences, or expectations, and attitudes regarding the procedure, rather than specifically those related to robotic-assisted surgery in the context of their surgery. *(c)* Editorials, case reports, conference abstracts, letters and reviews. *(d)* The search was limited to articles written in or translated into English or French.

### Article selection

Two independent investigators (AA and RA) screened the studies independently in three stages: first, following automated deduplication, the authors screened titles to remove ineligible abstracts. The titles and abstracts of all potentially relevant articles were then individually examined for suitability. Potentially eligible articles were obtained in full text and assessed for inclusion. Disagreements between the two original reviewers (AA and RA) were resolved by discussion with BJ and DH.

### Data extraction

Data were independently extracted onto MS Excel (Microsoft, California. USA) by *three* authors (AA, RA, BJ). A predesigned template was used to extract data on study characteristics (authors, year of publication, country, study design and type, number of patients, surgical specialty/s, robotic procedure/s, methodology including index measure used to assess patient satisfaction/expectations/attitudes/experiences/other and time points used, outcomes, length of follow-up), patient demographics and intraoperative and post-operative (mortality and complications) outcomes. Qualitative and quantitative outcomes were coded and recorded separately.

### Quality assessment

Quality appraisal was conducted independently for each full-text article. The two authors assessed the following study characteristics; study design, sampling strategy, participant recruitment methods, data collection (e.g., interview or survey methodologies), and completeness of data reporting. Potential biases were also considered in the context of RAS (e.g., variations in surgical expertise on the learning curve and patient demographics). A quality analysis was undertaken of all studies using the Critical Appraisal Skills Programme (CASP) Checklists for cross-sectional studies and qualitative data [12, 13].

### Data analysis

Data extraction sheets were imported into NVivo 12 for data management and analysis. An integrative approach to quantitative and qualitative data was employed using a sequential approach to data analysis [14, 15]. Quantitative data were transformed into qualitative data using the principles of thematic content analysis [16]. Quantitative data were coded to identify emerging concepts of interest. Codes reflecting similar findings and similar underlying constructs were grouped into categories. Categories were synthesized into themes if they were sufficiently similar. This approach enabled the summary of all evidence for a particular domain. A detailed codebook was created during the transcription, providing clear definitions of the codes to allow others to easily interpret and apply them to the raw data as needed. Following coding of the quantitative data, qualitative data were coded using the same principles. Qualitative data were coded into findings and categories, and the themes identified from the analysis of the quantitative data, or into new categories and themes if appropriate. This process facilitates comparison between findings from both quantitative and qualitative datasets, thereby appropriately triangulating and reviewing all emerging categories. The combined categories and themes were reviewed and synthesized by two reviewers (BJ/DH), with any discrepancies resolved by consensus.

## Results

**Fig. 1:**
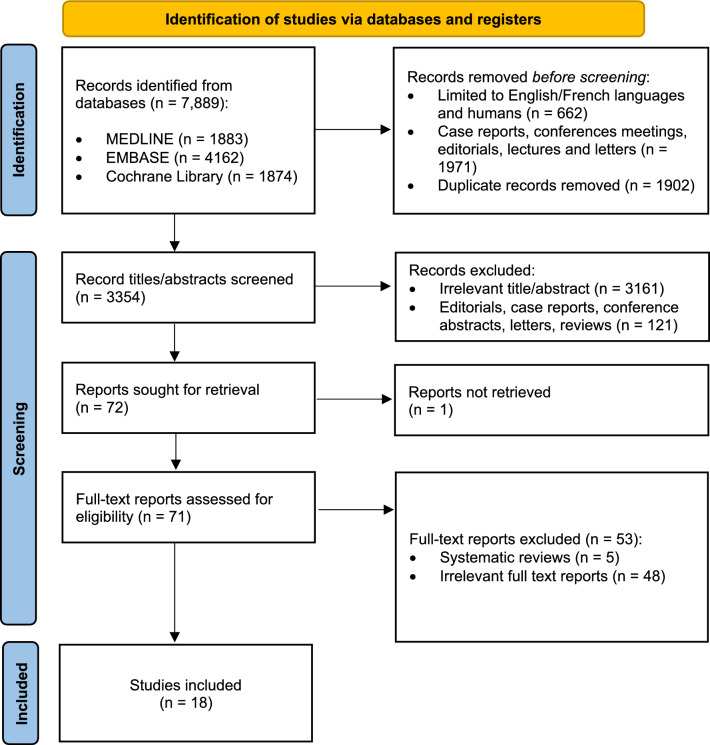
PRISMA flow diagram

The initial search identified a total of 7889 references; 1902 duplicates and 5916 inappropriate references were removed (Fig. 1). The remaining 71 full-text articles were retrieved and assessed for further evaluation, of which 18 met the eligibility criteria and were included in the final analysis.

### Study and baseline characteristics

Twelve quantitative studies, four qualitative studies and two mixed-methods studies were included in this review (Online Resource 2).

A combined total of 4,801 participants were approached across five specialities, and 4,033 (84%) were included in analyses following sufficient completion of the full assessment tools (Online Resources 2 and 3). This comprised 1244 members of the public (in four studies) and 2789 patients (in 15 studies), or 1973 (49%) males and 2016 (51%) females (Table 1). Overall, the median age at the time of completing questionnaires or participating in interviews was 56.6 years. The ethnic majority was Caucasian in nine studies, Hispanic in one study and not described in eight studies. Cohorts were assessed in relation to RAS procedures in gynecology (22%), colorectal (22%), urology (22%), orthopedics (22%), and cardiothoracic (17%) (Online Resource 2). Further patient demographics described are summarized in Table 1, such as education (56%), medical history (33%), and (if applicable) operative outcomes (11%).

**Table 1:** Participant characteristics

Authors, country	Participants approached	Participants included (n)	Age	Sex (n) M/F	Race/ Ethnicity	Language	Country	Education	Associated Diagnosis	Procedure	Cancer-related?
Ryan et al., USA [15]	150	89	N.R	52/37	N.R	English	USA	N.R	N.R	Robotic-assisted cardiothoracic surgery	No
El Douaihy et al., USA [26]	377	188	60	60/0	N.R	English	USA	N.R	Prostate cancer	Robotic-assisted radical prostatectomy	Yes
Dixon et al., Canada [29]	40	38	N.R	16/22	N.R	English	Canada	N.R	N.R	Partial colectomy (robot-assisted vs. conventional laparoscopic)	Yes
Herling et al., Denmark [21]	15	12	N.R	0/12	N.R	N.R	Denmark	N.R	Endometrial cancer	Robotic-assisted laparoscopic hysterectomy	Yes
Irani et al., USA [16]	242	219	36	0/219	Caucasian (33%)	English	USA	No minimum	N.R	N.R	N.R
Reynolds et al., Australia [28]	412	214	N.R	214/0	N.R	English	Australia	Most common level was university/ tertiary degree: 51.4%	Prostate cancer	Robotic-assisted radical prostatectomy	Yes
Chu et al., USA [17]	N.D	176	58	0/176	Caucasian (84%)	English	USA	Minimum of Bachelor’s degree (42%)	POP	POP repair	No
Stai et al., USA [24]	298	264	45	154/ 110	Caucasian (88%)	English	USA	Minimum of Bachelor’s degree (39%)	N/a (public)	N/a (public)	N/A (public)
Pagani et al., USA [18]	615	588	39	263/ 325	Caucasian (67.7%)	English (94.9%)	USA	No minimum	N/a (public)	N/a (public)	N/A (public)
Patel et al., Canada [30]	459	411	65	170/ 241	Caucasian (94%)	English	Canada	Minimum of Bachelor’s degree (20%)	Lung cancer	Robotic thoracic surgery	Yes
Muaddi et al., Canada [27]	362	362	53	232/ 130	218 white, 131 black or African American	English	Canada	N.R	N/a (public)	Comparison between laparoscopic surgery and robotic surgery	N/A
Claydon et al., UK [22]	89	27	71	18/9	N.R	English	UK	N.R	Colorectal cancer	Elective resection	Yes
Moloney et al., Ireland [7]	12	12	66	4/8	N.R	English	Ireland	N.R	N.R	N.R	N.R
Wu et al., China [23]	11	11	53	4/7	N.R	Mandarin	China	1/11 went to college or university	N.R	Multiple	N.R
Abdelaal et al., USA [19]	489	440	67	242/ 197	Caucasian (88%)	English	USA	Minimum of Bachelor’s degree (24%)	N.R	Total knee arthroplasty	No
Pinci et al., Puerto Rico [20]	602	580	51	268/ 312	Hispanics	Spanish	Puerto Rico	No minimum	N.R	Total joint arthroplasty	No
Chang et al., USA [8]	410	360	63	147/ 213	Caucasian (72.2%)	English	USA	No minimum	N.R	Reconstruction surgery (Hip/Knee)	No
Ashmore et al., UK [25]	12 patients, 30 non-patients	42 in total (12 qualitative, 30 quantitative	66	0/66	Phase 1: ND Phase 2: Group A 71.4–100% white	English	UK	N.R	N.R	Robotic-assisted gynecologic surgery	N.R

*N.R*. not reported, *POP* pelvic organ prolapse

Studies were primarily from the USA (44%), Canada (17%), the UK (11%), and other countries (28%). Study designs included 17 cross-sectional studies and 1 cohort study (Online Resource 2). Members of the public were sampled through online and digital recruitment with small compensation schemes (Online Resource 3). Studies used a variety of methods to assess patient and public perceptions of RAS, including self-administered questionnaires (61%), semi-structured interviews (28%), and telephone-administered survey questionnaires (11%) (Online Resource 3). Overall, the quality of the qualitative studies included in this review was of good quality, with 70–90% compliance with the CASP checklists (Online Resource 4). Quantitative studies were of heterogeneous quality, ranging from 50 to 80%.

### Identified themes

**Fig. 2:**
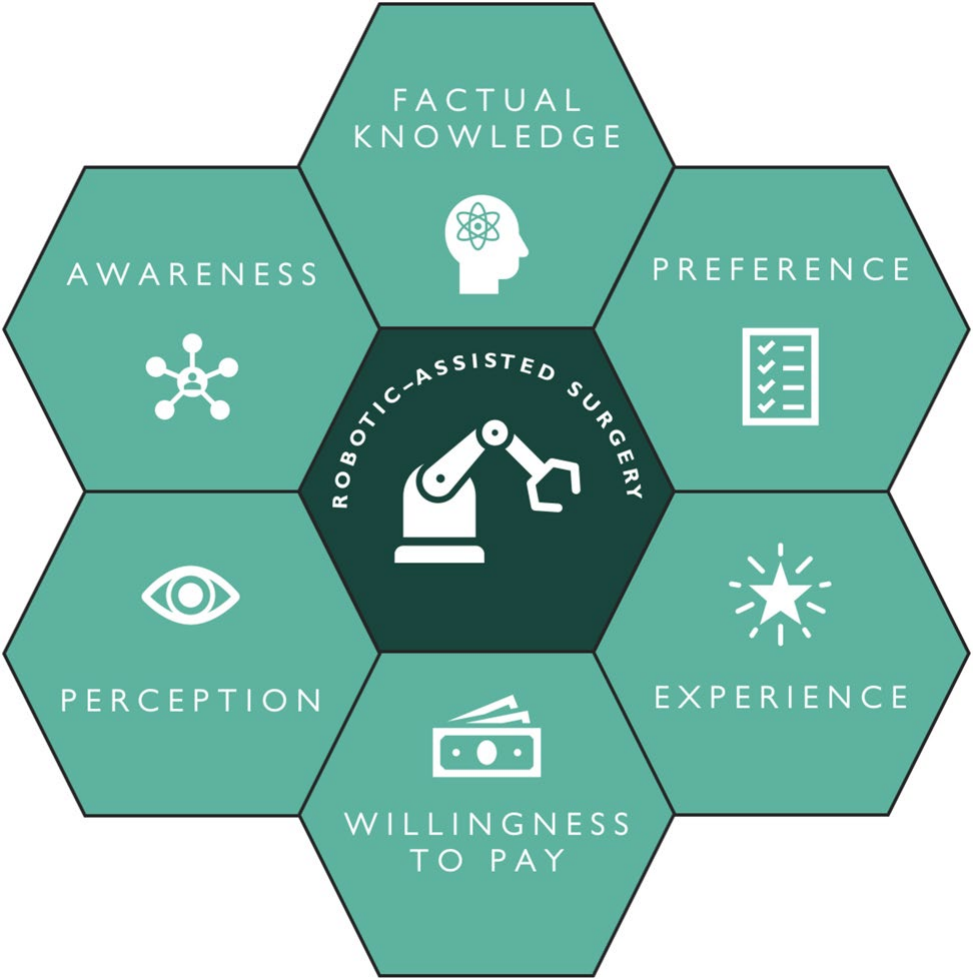
Identified themes of patient and public perspectives on robotic-assisted surgery

Six themes (factual knowledge and understanding, awareness, perception and expectations, patient preferences, experiences and satisfaction, and willingness to pay) were identified (Fig. 2) from the quantitative (*n* = 12) studies (Online Resource 5). Using the principles of content analysis, 85 findings were identified, resulting in 31 categories and six themes across the qualitative (*n* = 4) and mixed methods (*n* = 2) studies (Online Resource 6).

### Factual knowledge & understanding

Seven quantitative [8, 17–22] and all qualitative studies [7, 23–25] reported on participants’ baseline knowledge and understanding of RAS. Quantitative analyses reported misinterpretations of the role of the surgeon and robotic platform/console in RAS, and this was echoed in the qualitative narratives. Up to 67.5% of participants did not know or understand the premise of robotic surgery, including how the robotic platform works. Between 10 and 30% of participants believed that the robot was completely autonomous [8, 19, 20, 22]. Misconceptions were particularly prevalent among members of the public [20], and patients with a lower educational background and those without prior surgical experience [18].

### Awareness

Twelve studies commented on the awareness of participants at the time of study [7, 8, 18, 20–27]. Awareness was generally reported as very low, or superficial, and studies reported that 30–40% of participants had never heard of RAS [21, 22]. The primary information sources cited were television and online platforms [7, 20, 22, 25]. Previous surgical experience and healthcare exposure increased awareness (*p* < 0.05) [18, 22]. Some studies found that patients were unaware that RAS was an available option until it was offered to them by their healthcare professional [18, 24, 27].

### Perception and expectations

The perception and expectations of participants toward RAS were commented on by 16 studies [7, 8, 18–26, 26–30]. Participants perceived robotic surgery as being more precise, less invasive, and associated with better outcomes [8, 19, 20, 23, 28]. There were high expectations for faster recovery and fewer complications (*p* < 0.001) [19]. However, participants lacked trust in the robots in several studies [7, 24, 25], with one study reporting that 72.1% of participants believed robotic surgery had a higher chance of surgical error, compared to laparoscopic surgery [29]. Participants were generally apprehensive about RAS with concerns regarding robot malfunction, or misconceptions about the lack of a surgeon’s role in the operation [7, 20, 21, 25, 29]. Older members of the public were more likely to respond feeling “uncomfortable” about the concept of RAS (*p* < 0.05) [26]. Video-based interventions, such as pre-operative recordings and information videos, were associated with improved understanding, and realistic expectations in patients [27, 28].

### Patient preference

Eleven studies reported on participants’ preferences comparing RAS to other surgical approaches [7, 18–24, 27, 29, 31]. Three studies reported that participants preferred RAS due to expectations of improved recovery and reduced pain [20, 22, 23]. Other studies reported no preference, or preferences which were primarily influenced by provider or surgeon recommendation [7, 18, 19, 21, 24, 27]. Muaddadi et al*.* [29] reported that laparoscopic surgery was preferred by 62% of members of the public despite robotic surgeons perceived as being more competent (55.2%) and trustworthy (53.5%). One study reported a 43.7% increase (from 31.3 to 75.0%) in participants who would choose RAS over laparoscopic or open surgery, after watching an information video versus traditional pre-operative counseling [27]. Dixon et al*.* reported that marketing language, such as “state of the art” and “innovative” significantly (*p* < 0.001) increased the likelihood of patients choosing robot-assisted surgery, regardless of clinical evidence [31].

### Experiences and satisfaction

The patient experience of RAS was reported by all eight studies that used post-operative time point measures [7, 17, 23–25, 28, 30, 32]. Satisfaction with RAS was generally very high across studies, with 85%—98% patients reporting benefits of undergoing RAS [17, 28, 30, 32]. In study groups where patients viewed pre-operative explanatory videos or structured programs, post-operative satisfaction rates were higher [27, 28]. Qualitative insights further expanded on these results and reported that communication, emotional reassurance, and wider care from clinical staff increased overall experience and satisfaction [7, 23, 24, 30]. Where dissatisfaction occurred post-operatively, it was generally due to unmet pre-operative expectations or unexpected post-operative complications, but not explicitly attributed to robotic surgery itself [7].

### Willingness to pay

Willingness to pay (WTP) for RAS was commented on by three studies based in the USA, Canada, and Australia [21, 30, 32]. In one study, 81% of patients were willing to pay out of pocket for robotic thoracic surgery. This was positively associated with higher income and positive post-operative experiences (*p* < 0.001) [32]. WTP was influenced by the surgeons’ advocacy for RAS [21].

## Discussion

The rapid advancement of RAS has transformed modern surgical practice; yet this systematic review suggests that patient and public understanding and perception of RAS technology are complex and variable. While the adoption of robotic surgery continues to expand globally, our review highlights a substantial spectrum of misconceptions among patients and the public as documented across the literature.

A central theme across studies is the widespread belief that robots perform surgery autonomously with minimal or no human oversight [5, 8, 19, 20, 23]. This misunderstanding, reported in up to one-third of patients by Chu et al*.* [19] and members of the public by Pagani et al*.* [20], fuels anxiety about safety, control, and technological reliability, and reflects a failure to communicate sufficient information about how robotic surgical systems work and the surgeon’s role in maintaining complete control of the console [33, 34]. Despite this, patients frequently associate RAS with advanced precision, minimal scarring, and a faster recovery [8, 19, 20, 22, 24, 30]. Although some of these perceptions are supported by clinical evidence [1, 35, 36], including a 2024 comparative umbrella review of the clinical effectiveness of robotic versus laparoscopic and open surgery [2], many patient expectations are overly generalized and lack nuance. Conversely, significant concerns persist among patients regarding intraoperative safety risks. Muaddi et al*.* found that over 70% of surveyed members of the public expressed concerns about robot malfunctions causing internal damage [29], while Pagani et al*.* found that 28% worried about loss of surgeon control [20]. While these fears often contradict current real-world safety data [37, 38] and established protocols, they reflect a genuine public unease with the integration of technology into intimate, high-stakes environments, such as surgery. This is not unique to RAS because mistrust of innovation is a common phenomenon seen in healthcare [39]. The contrasting patient attitudes toward RAS, particularly in patients who underwent robotic surgery, also reflect a lack of fully informed decision-making as found in previous studies [40, 41]. Qualitative data echoed this and explain why, despite believing RAS offered greater precision, patients preferred laparoscopic or open surgery for perceived greater surgeon involvement, even post-operatively [24].

Patient awareness of RAS is frequently shaped by traditional media portals and social media platforms than by formal healthcare communication [42]. Older adults and those with lower levels of education reported lower exposure to accurate information, and often rely on second-hand or misleading sources [25, 26]. Younger adults, conversely, tend to exhibit more favorable and comfortable attitudes toward robotic surgery [26]. This may reflect generational differences in technology acceptance and the sources through which patients learn about robotic surgery. Social media has emerged as a primary information source for surgery among the majority of patients [43, 44]. While this highlights the powerful role of digital communication in healthcare awareness, it also raises concerns about the accuracy and completeness of information patients receive through these channels. A recent study found that more than a third (35%) of the examined social media videos on surgical procedure topics contained misinformation [43]. A plausible explanation for the patient’s use of social media is the lack of comprehensive, accessible, and trusted information within existing healthcare systems. It reinforces the need for institutions to develop and disseminate accurate, engaging, and patient-friendly educational materials that address common misconceptions while grounding expectations in clinical reality. Furthermore, Dixon et al*.* found that marketing language significantly (*p* < 0.001) increases the likelihood that patients will choose RAS, regardless of clinical evidence [31]. Terms like “innovative” or “cutting-edge” may inadvertently bias patients toward robotic approaches, even in the absence of strong comparative evidence. This highlights the ethical implications of how RAS is communicated to the public and underscores the importance of providing neutral and balanced, evidence-based patient education to facilitate shared decision-making.

Education is a powerful tool for bridging the information divide and improving decision-making. Studies employing video-based or animated educational interventions demonstrated improved patient understanding, increased satisfaction, and the building of more realistic expectations [27, 28]. One study reported that educational videos significantly increased patient preference for RAS, underscoring the persuasive impact of targeted, multimodal education [27]. There is growing evidence that VR-based pre-operative education improves patient understanding, satisfaction, and significantly reduces pre-operative anxiety compared to standard care [45–47]. These studies show promise in improving patient understanding of robotic surgical procedures. However, further large-scale studies are needed to confirm these benefits and explore their impact on clinical outcomes. The goal should be to provide patients with sufficient information to make informed decisions while addressing the specific fears and misconceptions that currently influence patient attitudes toward robotic surgery. Postoperative satisfaction was closely linked to how well outcomes aligned with pre-operative expectations [7]. When patients are well-informed and experience matches expectations, satisfaction is typically high [27].

This review is subject to several limitations. First, the studies included were heterogeneous in design, quality, and methodology. Most were cross-sectional, retrospective, and based on self-reported data, which limits the ability to draw causal inferences or assess changes in attitudes over time (Online Resource 2). However, the mixed-methods synthesis enabled the identification of overlapping and complementary perspectives across data. Only a minority of studies included longitudinal follow-up, making it challenging to explore how perceptions may evolve before and after surgery. The completeness of data was variable, with six studies reporting response rates below 60% or not at all (Table 1), raising concerns about non-response and selection bias. It is possible that individuals with stronger views were more likely to participate, potentially skewing the results. Geographically, the majority of participants came from high-income, English-speaking countries, particularly the USA and Canada, which limits the generalisability and applicability of the findings, such as willingness to pay or access to information, to global populations or lower-income settings (Table 1). Sociocultural, economic, and healthcare system differences likely shape perceptions of robotic-assisted surgery in ways that are not captured by the current literature. Additionally, the definitions and assessment tools used to measure key constructs such as “preference,” “understanding,” or “satisfaction” varied widely, introducing further heterogeneity in outcome reporting. Few studies explicitly addressed the impact of health literacy, digital literacy, or language barriers on participants’ responses. Finally, although this review employed a robust mixed-methods synthesis approach, the findings remain dependent on the quality and scope of the included literature.

There are several actionable recommendations for clinical teams, institutions, and policymakers. Patient education initiatives should prioritize delivering accurate and balanced information about robotic surgery, especially concerning the surgeon’s role, risks, and benefits. Resources should be created in various formats, including visual or video content, and tailored to different levels of health literacy and digital access. Additionally, marketing and promotional language used by providers should be reviewed to ensure it does not bias patient choices or undermine informed consent. Future research should adopt prospective, multicentre designs and explore educational interventions such as virtual reality or interactive platforms that directly address misconceptions, promote equity in access, and improve informed decision-making. Studies should also examine whether speciality-specific consultation practices and styles influence patient understanding and satisfaction. Although the studies included in this review were not limited by time, the publication period (2020–2024) was relatively narrow due to the recent growth of RAS. No significant shifts in public perception were observed during this timeframe; future research could explore how perceptions evolve as the technology becomes more widely adopted globally.

## Conclusion

Patients and the public hold diverse views on robotic-assisted surgery with multiple factors contributing to these views and various misconceptions. Ultimately, this review underscores the importance of centering the patient voice in the ongoing integration of RAS into surgical care. As robotic technology continues to evolve, so too must our approaches to patient engagement. Digital education tools, visual learning resources, and transparent communication about both capabilities and limitations of robotic technology show promise for building appropriate patient expectations and trust. Transparent communication, personalized education, and neutral framing of robotic technologies will be essential to empower patients and support shared, evidence-based decision-making in the era of robotic healthcare.

## Supplementary Information

Below is the link to the electronic supplementary material.

Supplementary file1 (DOCX 24 KB)

Supplementary file2 (DOCX 32 KB)

Supplementary file3 (DOCX 30 KB)

Supplementary file4 (DOCX 28 KB)

Supplementary file5 (DOCX 35 KB)

Supplementary file6 (DOCX 24 KB)

## Data Availability

No datasets were generated or analyzed during the current study.

## Abbreviations

CASP: Critical appraisal skills program checklist
MeSH: Medical subject headings
PICOS: Population, intervention, comparison, outcomes, and study design
PRISMA: Preferred reporting items for systematic reviews and meta-analyses
RAS: Robotic-assisted surgery
WTP: Willingness to pay
