# Supplementary material for: From expectations to experiences: a systematic review of patient and public perspectives on robotic surgery
Source: J Robot Surg. 2025 Aug 14;19(1):484. doi: 10.1007/s11701-025-02649-y (PMC12354569; doi:10.1007/s11701-025-02649-y)
Supplement: Supplementary file 1 — Supplementary file1 (DOCX 24 KB) [file 11701_2025_2649_MOESM1_ESM.docx]

**Supplementary Information**

**From Expectations to Experiences: A Systematic Review of Patient Perspectives on Robotic Surgery**

*B Jauniaux^1^, A Anand^2^, R Abbas^2^, DP Harji^1,3,4^*

Benoit Jauniaux*,* ^1^Department of Colorectal Surgery, Manchester University NHS Foundation Trust, Manchester, UK*;* [benoit.jaunaux@doctors.org.uk](mailto:benoit.jaunaux@doctors.org.uk), ORCID ID 0000-0002-2527-2112

Ajitesh Anand, ^2^University of Manchester, Manchester, UK; ajitesh.anand@doctors.org.uk, ORCID ID 0000-0003-0184-841X

Rahma Abbass, ^2^University of Manchester, Manchester, UK; [rahmaaabbas02@gmail.com](mailto:rahmaaabbas02@gmail.com), ORCID ID 0009-0002-7966-5277

Deena Harji*,* ^1^Department of Colorectal Surgery, Manchester University NHS Foundation Trust, Manchester, UK, ^4^Robotics and Digital Surgery Initiative, Royal College of Surgeons of England, England, ^5^ Clinical Trials Research Unit, Leeds Institute of Clinical Trials Research, University of Leeds, Leeds, UK; d.harji@leeds.ac.uk ORCID ID 0000-0002-8493-3312

**Corresponding author:** Deena Harji, ^1^Department of Colorectal Surgery, Manchester University NHS Foundation Trust, Manchester, UK, ^4^Robotics and Digital Surgery Initiative, Royal College of Surgeons of England, England, ^5^Clinical Trials Research Unit, Leeds Institute of Clinical Trials Research, University of Leeds, Leeds, UK; d.harji@leeds.ac.uk

**Table S1. Medline and Cochrane Library Search Strategy**

**1.** ("robot*" or "robotic surg*" or "robot-assisted surg*" or "robotic-assisted" or "robotic surgical procedure*" or "robot* procedure*").ab,ti.

**2.** Robotic Surgical Procedures/

**3.** 1 or 2

**4.** ("surg*" or "cardiac surg*" or "cardiothoracic surg*" or "heart surg*" or "chest surg*" or "abdom* surg*" or "thoracic surg*" or "urolog* surg*" or "colorect* surg*" or "colon surg*" or "cosmetic surg*" or "orthop?edic surg*" or "plastic surg*" or "reconstruct* surg*" or "ENT surg*" or "ear nose throat surg*" or "otolaryng* surg*" or "head and neck surg*" or "vascular surg*" or "transplant* surg*" or "p?ediatric surg*" or "maxillofacial surg*" or "ophthalm* surg*" or "trauma surg*" or "hepatobiliary surg*" or "endocrine surg*" or "thyroid surg*" or "adrenal surg*").ab,ti.

**5.** Specialties, Surgical/

**6.** 4 or 5

**7.** (Appendectom* or Pancreatectom* or Esophagectom* or "Bariatric Surgery" or "Gastric bypass" or "Sleeve gastrectom*" or "Laparoscopic adjustable gastric band" or "Hernia repair" or Splenectom* or Cholecystectom* or "Cervical cerclage" or Prostatectom* or Nephrectom* or Cystectom* or Pyeloplast* or Ureteroscop* or "Lung resection" or Esophagectom* or Thymectom* or Mediastinoscop* or "Coronary artery bypass graft*" or "CABG" or "Mitral valve repair" or "Atrial fibrillation ablation" or "Aortic valve replacement" or Laryngectom* or Tympanoplast* or Parotidectom* or Adenoidectom* or "Hip arthroplast*" or "Knee arthroplast*" or "Shoulder surgery").ab,ti.

**8.** Surgical Procedures, Operative/

**9.** 7 or 8

**10.** ("patient* perspective*" or "patient* view*" or "patient* opinion*" or "patient* attitude*" or "patient* outlook*" or "patient* perception*" or "patient* standpoint*" or "patient* insight*" or "patient* preference*" or "patient* belief*" or "patient* understanding*" or "patient* judgment*" or "patient* expectation*" or "patient* anticipation*" or "patient* hope*" or "patient* prediction*" or "patient* expectancy*" or "patient* experience*" or "patient* journey*" or "patient* encounter*" or "patient* feedback*" or "patient* observation*" or "patient* narrative*" or "patient* report*" or "patient* account*" or "patient* stor*" or "patient* satisfaction*" or "patient* interaction*" or "patient* participation*" or "patient* involvement*" or "patient* engagement*").ab,ti.

**11.** Patient Satisfaction/

**12.** *Attitude to Health/

**13.** 10 or 11 or 12

**14.**  6 or 9

**15.**  3 and 13 and 14

**Table S2. Embase Search Strategy**

**1.** ("robot*" or "robotic surg*" or "robot-assisted surg*" or "robotic-assisted" or "robotic surgical procedure*" or "robot* procedure*").ab,ti.

**2.** robotics/ or robot assisted surgery/ or robotic surgical system/

**3.** ("surg*" or "cardiac surg*" or "cardiothoracic surg*" or "heart surg*" or "chest surg*" or "abdom* surg*" or "thoracic surg*" or "urolog* surg*" or "colorect* surg*" or "colon surg*" or "cosmetic surg*" or "orthop?edic surg*" or "plastic surg*" or "reconstruct* surg*" or "ENT surg*" or "ear nose throat surg*" or "otolaryng* surg*" or "head and neck surg*" or "vascular surg*" or "transplant* surg*" or "p?ediatric surg*" or "maxillofacial surg*" or "ophthalm* surg*" or "trauma surg*" or "hepatobiliary surg*" or "endocrine surg*" or "thyroid surg*" or "adrenal surg*").ab,ti.

**4.** surgery/

**5.** (Appendectom* or Pancreatectom* or Esophagectom* or "Bariatric Surgery" or "Gastric bypass" or "Sleeve gastrectom*" or "Laparoscopic adjustable gastric band" or "Hernia repair" or Splenectom* or Cholecystectom* or "Cervical cerclage" or Prostatectom* or Nephrectom* or Cystectom* or Pyeloplast* or Ureteroscop* or "Lung resection" or Esophagectom* or Thymectom* or Mediastinoscop* or "Coronary artery bypass graft*" or "CABG" or "Mitral valve repair" or "Atrial fibrillation ablation" or "Aortic valve replacement" or Laryngectom* or Tympanoplast* or Parotidectom* or Adenoidectom* or "Hip arthroplast*" or "Knee arthroplast*" or "Shoulder surgery").ab,ti.

**6.** surgical technique/

**7.** 1 or 2

**8.** 3 or 4

**9.**  5 or 6

**10.** ("patient* perspective*" or "patient* view*" or "patient* opinion*" or "patient* attitude*" or "patient* outlook*" or "patient* perception*" or "patient* standpoint*" or "patient* insight*" or "patient* preference*" or "patient* belief*" or "patient* understanding*" or "patient* judgment*" or "patient* expectation*" or "patient* anticipation*" or "patient* hope*" or "patient* prediction*" or "patient* expectancy*" or "patient* experience*" or "patient* journey*" or "patient* encounter*" or "patient* feedback*" or "patient* observation*" or "patient* narrative*" or "patient* report*" or "patient* account*" or "patient* stor*" or "patient* satisfaction*" or "patient* interaction*" or "patient* participation*" or "patient* involvement*" or "patient* engagement*").ab,ti.

**11.** patient satisfaction/ or patient attitude/

**12.** 8 or 9

**13.** 10 or 11

**14.** 7 and 12 and 13
