# Supplementary material for: From expectations to experiences: a systematic review of patient and public perspectives on robotic surgery
Source: J Robot Surg. 2025 Aug 14;19(1):484. doi: 10.1007/s11701-025-02649-y (PMC12354569; doi:10.1007/s11701-025-02649-y)
Supplement: Supplementary file 2 — Supplementary file2 (DOCX 32 KB) [file 11701_2025_2649_MOESM2_ESM.docx]

**Supplementary Information**

**From Expectations to Experiences: A Systematic Review of Patient Perspectives on Robotic Surgery**

*B Jauniaux^1^, A Anand^2^, R Abbas^2^, DP Harji^1,3,4^*

Benoit Jauniaux*,* ^1^Department of Colorectal Surgery, Manchester University NHS Foundation Trust, Manchester, UK*;* [benoit.jaunaux@doctors.org.uk](mailto:benoit.jaunaux@doctors.org.uk), ORCID ID 0000-0002-2527-2112

Ajitesh Anand, ^2^University of Manchester, Manchester, UK; ajitesh.anand@doctors.org.uk, ORCID ID 0000-0003-0184-841X

Rahma Abbass, ^2^University of Manchester, Manchester, UK; [rahmaaabbas02@gmail.com](mailto:rahmaaabbas02@gmail.com), ORCID ID 0009-0002-7966-5277

Deena Harji*,* ^1^Department of Colorectal Surgery, Manchester University NHS Foundation Trust, Manchester, UK, ^4^Robotics and Digital Surgery Initiative, Royal College of Surgeons of England, England, ^5^ Clinical Trials Research Unit, Leeds Institute of Clinical Trials Research, University of Leeds, Leeds, UK; d.harji@leeds.ac.uk ORCID ID 0000-0002-8493-3312

**Corresponding author:** Deena Harji, ^1^Department of Colorectal Surgery, Manchester University NHS Foundation Trust, Manchester, UK, ^4^Robotics and Digital Surgery Initiative, Royal College of Surgeons of England, England, ^5^Clinical Trials Research Unit, Leeds Institute of Clinical Trials Research, University of Leeds, Leeds, UK; d.harji@leeds.ac.uk

**Table S3. Study Characteristics**

| Author(s), country | Year | RAS specialty | Robotic device | Study design | Analysis | Relevant research question | Public / patients | Listed inclusion criteria (or *exclusion* criteria) | Participants details | Summary of findings |
| --- | --- | --- | --- | --- | --- | --- | --- | --- | --- | --- |
| Ryan et al., USA | 2009 | Cardiothoracic | Da Vinci | Cross-sectional | Quantitative | 1. Evaluate patient's perceptions and knowledge after robotic-assisted cardiothoracic surgery | Patients | (1). All patients undergoing robotic-assisted cardiothoracic surgery (Feb 2002-June 2007) by 2 surgeons. | Patients who underwent robotic-assisted cardiothoracic. | § 96.6% of patients were satisfied with the information provided by their surgeon. § 92.1% felt they understood the information as it was described to them. |
| El Douaihy et al., USA | 2010 | Urology | N.D. | Retrospective cohort study | Quantitative | 1. Assess patient expectations and satisfaction following RARP. | Patients | (1). Patients who underwent RARP by a single surgeon (AT) at the institution. (2). Patients who participated in the structured preoperative education and counselling program. | All were patients who underwent Robotic-assisted radical prostatectomy (RARP). | § Overall satisfaction 93% of patients were satisfied, 0.5% regretted having surgery. § 97% considered oncologic clearance as the most important goal. § Patients with biochemical recurrence (PSA > 0.2 ng/mL) were more likely to be dissatisfied. |
| Dixon et al., Canada | 2012 | Colorectal | N.D. | Cross-sectional | Quantitative | 1. Determine how marketing language influences patient preference for RAS vs. conventional laparoscopic surgery in a hypothetical colon cancer diagnosis. | Patients | (1). English-speaking adults (aged 18–75 years). (2). Patients attending an ambulatory general surgery clinic. (3). Able to complete a survey assessing surgical preferences. | All participants were patients attending a general surgery clinic. | § Marketing language significantly influenced patient preference for robotic-assisted surgery. § Under the marketing frame: 52.6% (20/38) chose robotic-assisted surgery. § Under the evidence-based frame: 31.6% of those who initially chose robotic-assisted surgery switched to laparoscopic surgery (P = 0.005). § Older patients were more likely to be influenced by marketing language (odds ratio: 4.8, P = 0.04). |
| Herling et al., Denmark | 2015 | Gynaecology | N.D. | Cross-sectional | Qualitative | 1. Explore experiences RAS (hysterectomy in early-stage endometrial cancer). | Patients | (1). Endometrial cancer at final histology.  (2). Sufficient language proficiency in Danish. | All were patients who underwent robotic-assisted laparoscopic hysterectomy. | § "Surgery was a piece of cake" – Women generally felt robotic surgery was safe and precise, but they lacked understanding of the technical details. § "Preparing oneself by seeking information" – Women sought extensive information online and from healthcare providers, but still felt uncertain about postoperative expectations and anatomical changes. |
| Irani et al., USA | 2016 | Gynaecology | N.D. | Cross-sectional | Quantitative | 1. Knowledge regarding robotic-assisted surgery. 2. Factors that affect patients' knowledge of surgical approaches. | Patients | (1). Women receiving routine OBGYN care. | Patients at a community hospital clinic and hospital-affiliated private office. | § Factors associated with greater understanding are previous abdo sx and higher educational level |
| Reynolds et al., Australia | 2018 | Urology | N.D. | Cross-sectional | Mixed | 1. Determine patient satisfaction and experience after RARP. | Patients | (1). Patients that had undergone RARP by 2 surgeons (Jan 2014 - June 2016). | All were patients who underwent RARP. | § 97.6% of patients were 'satisfied' or 'extremely satisfied' with their experience           § Key factors influencing satisfaction: - Quality and timing of information received. - Postoperative recovery experience (continence, pain management, and mobility). |
| Chu et al., USA | 2019 | Gynaecology | N.D. | Cross-sectional | Quantitative | 1. Compare patient perception of robotic surgery to other surgical approaches for POP. 2. Evaluate general understanding of robotic surgery. | Patients | (1). Women diagnosed with new pelvic organ prolapse (POP).  (2). No previous surgical counselling. | New patients presenting with POP. Subjects had no prior surgical counselling. | § Most had no surgical preference (66.3%). § Knowledge about robotic surgery was low, even among subjects who expressed preference. |
| Stai et al., USA | 2020 | N/A | N.D. | Cross-sectional | Quantitative | 1. Understand better the public perception of AI and robotic surgery. | Public | (1). >18 y/o, who volunteered to complete the survey at a state fair. | Attendees at a Minnesota State Fair. | § Overestimation of % of robotic surgery that is autonomous. § 55% uncomfortable with autonomous robotic surgery. |
| Pagani et al., USA | 2021 | Orthopaedics | N.D. | Cross-sectional | Quantitative | 1. Examine public perceptions of orthopaedic RAS | Public | (1). >18 y/o, residing in US w/ valid SSN. (2). Participants on Amazon Mechanical Turk (online survey platform). | Surveys of Amazon Mechanical Turk workers. | § Participants expect good outcomes of robotic surgery including better results, fewer complications, less pain and faster recovery. § The outcomes of the study are limited by the public's misperception of the robot's role. |
| Patel et al., Canada | 2022 | Cardiothoracic | N.D. | Cross-sectional | Quantitative | 1. Patient-reported satisfaction with Robotic Thorascopic Surgery (RTS). | Patients | (1). All patients who underwent RTS for early-stage lung cancer. | Patients who underwent robotic-assisted thoracic surgery. | § 86% rated their overall experience with RTS rated as 8 or higher out of 10 (10 = excellent). |
| Muaddi et al., Canada | 2022 | N/A | N.D. | Cross-sectional | Quantitative | 1. Investigate the public's perception of robotic surgery | Public | (1).English-speaking. (2). >50 y/o. (3). Living in Canada or USA. | General public. | § Fear of Outcomes: 78.2% of respondents feared outcomes of robotic surgery, compared to 14.9% for laparoscopic surgery (p < 0.001). § Procedure Preference: 64.4% preferred laparoscopic surgery over robotic surgery (35.6%) (p < 0.001). |
| Claydon et al., UK | 2023 | Colorectal | N.D. | Cross-sectional | Qualitative | 1. Investigate perceptions of RAS among patients. 2. Determine salient factors affecting patients' perioperative experience. | Patients | *Exclusion:*  (1). < 18 y/o. (2). Lacking capacity to consent. (3). Locally advanced or recurrent rectal ca. (4). Resections with flap reconstruction. (5). Pts not understanding English. | Patients undergoing elective resection of L colorectal cancer. | § Patients understood technological benefits of RAS but concerned by risk of tech failure. § All groups concerned regarding complications and experience affected emotionally (due to lack of preparation for significant factors). |
| Moloney et al., Ireland | 2023 | Colorectal, Urology | N.D. | Cross-sectional | Qualitative | 1. Explore patient-reported experiences of RAS. | Patients | (1). >18 y/o. (2). Undergone RAS within the previous 6 weeks. | Patients who underwent robotic surgery. | § Participant's experienced a lack of pre-operative preparation, resulting in feelings of anxiety and some negative perceptions of RAS. |
| Wu et al., China | 2023 | Cardiothoracic, Colorectal, Urology | Da Vinci | Cross-sectional | Qualitative | 1. Understand the experience of patients undergoing Da Vinci robotic surgery | Patients | (1). Patients who chose robotic surgery. (2). Satisfactory capacity, able to read & write. | Patients who underwent Da Vinci robotic surgery. | § Patients had a strong desire for information about the surgery and sought information about it in a variety of ways, but had insufficient understanding of the surgical methods. |
| Abdelaal et al., USA | 2023 | Orthopaedics | N.D. | Cross-sectional | Quantitative | 1. Understand how patients view the role of robotics during total knee arthroplasty (TKA). | Patients | (1). >18 y/o. (2). Patients presenting for consultation for TKA between 2021-2022. (3). No cognitive impairment. | Potential robotic surgery patients at the time of their surgery scheduling. | § 40% of patients had no knowledge regarding robotic surgery. Many had various concerns. |
| Pinci et al., Puerto Rico | 2024 | Orthopaedics | N.D. | Cross-sectional | Quantitative | 1. Knowledge and perspectives of Hispanics regarding total joint arthroplasty (TJA). 2. Factors affecting preferences and decisions re rTJA. | Patients | (1). > 21 y/o. (2). Hispanics. (3). Completed survey with informed consent. | Patients at clinics encompassing surgical and nonsurgical specialties at a major tertiary medical centre. | § Less than half familiar with robotic surgery. § Around half of the group preferred robotic surgery due to expected better outcomes and faster recovery. § Further patient education required to assist informed decisions and associated clinical outcomes. |
| Chang et al., USA | 2024 | Orthopaedics | N.D. | Cross-sectional | Quantitative | 1. Investigate effect of patient demographics on interest in robotic TJA. 2. Patient perceptions re rTJA. | Patients | (1). >18 y/o presenting for initial hip/knee reconstruction consultation prior to seeing provider. | Patients at an orthopaedic clinic. | § Patient interest and understanding of rTJA varies. |
| Ashmore et al., UK | 2024 | Gynaecology | N.D. | Cross-sectional (two separate phases) | Mixed | 1. Investigate patient's understanding and perception of gynae RAS. 2. Determine whether the format of RAS information resources influences acceptability of RAS. | Both patients & public | (1) Patients who had undergone gynae RAS procedure. (2) >18 y/o. (3). Satisfactory capacity, able to read & write. | 12 patients who underwent gynae RAS and 30 female members of general public who had not. | § Lack of knowledge increases anxiety and influences acceptability of RAS. § Acceptance of RAS increases after provision of information, 76.7% of 30 women preferring video resources. § Written information leaflets were often insufficient, prompting patients to seek info from alternative sources. |
