# Supplementary material for: From expectations to experiences: a systematic review of patient and public perspectives on robotic surgery
Source: J Robot Surg. 2025 Aug 14;19(1):484. doi: 10.1007/s11701-025-02649-y (PMC12354569; doi:10.1007/s11701-025-02649-y)
Supplement: Supplementary file 3 — Supplementary file3 (DOCX 30 KB) [file 11701_2025_2649_MOESM3_ESM.docx]

**Supplementary Information**

**From Expectations to Experiences: A Systematic Review of Patient Perspectives on Robotic Surgery**

*B Jauniaux^1^, A Anand^2^, R Abbas^2^, DP Harji^1,3,4^*

Benoit Jauniaux*,* ^1^Department of Colorectal Surgery, Manchester University NHS Foundation Trust, Manchester, UK*;* [benoit.jaunaux@doctors.org.uk](mailto:benoit.jaunaux@doctors.org.uk), ORCID ID 0000-0002-2527-2112

Ajitesh Anand, ^2^University of Manchester, Manchester, UK; ajitesh.anand@doctors.org.uk, ORCID ID 0000-0003-0184-841X

Rahma Abbass, ^2^University of Manchester, Manchester, UK; [rahmaaabbas02@gmail.com](mailto:rahmaaabbas02@gmail.com), ORCID ID 0009-0002-7966-5277

Deena Harji*,* ^1^Department of Colorectal Surgery, Manchester University NHS Foundation Trust, Manchester, UK, ^4^Robotics and Digital Surgery Initiative, Royal College of Surgeons of England, England, ^5^ Clinical Trials Research Unit, Leeds Institute of Clinical Trials Research, University of Leeds, Leeds, UK; d.harji@leeds.ac.uk ORCID ID 0000-0002-8493-3312

**Corresponding author:** Deena Harji, ^1^Department of Colorectal Surgery, Manchester University NHS Foundation Trust, Manchester, UK, ^4^Robotics and Digital Surgery Initiative, Royal College of Surgeons of England, England, ^5^Clinical Trials Research Unit, Leeds Institute of Clinical Trials Research, University of Leeds, Leeds, UK; d.harji@leeds.ac.uk

**Table S4. Methodological Design of Included Studies**

| **Author(s), country** | **Quest-ions (n)** | **Format** | **Sampling Strategy** | **Recruit-ment** | **Data Collection** | **Time-points** | **Total time-points** | **Pre-operative time-points?** | **Post-operative time-points?** | **Length of follow up** | **Analysis method (Qualitative only)** | **Statistics (Quantitative only)** |
| --- | --- | --- | --- | --- | --- | --- | --- | --- | --- | --- | --- | --- |
| Ryan et al., USA | 12 | Telephone-administered survey questionnaire | All eligible patients | N.R. | Questionnaire asked by interviewers | Single | 1 | N/A | Between 1-12 months post-op (median 84.6 days) | 84.6 days (median) | N/A | Student’s t-test, Pearson’s χ² test Mann-Whitney U test, p < 0.05 considered statistically significant. |
| El Douaihy et al., USA | 6 | In person (post-op) self-administered survey questionnaire | N.R. | N.R. | Paper questionnaire | Single | 1 | N/A | Median of 4.8 months | 4.8 months (median, range 1–10 months) | N/A | Chi-squared tests, ANOVA, Fisher’s exact test, and Pearson’s correlation were used to analyze associations. |
| Dixon et al., Canada | N.R. | In person (clinic) self-administered survey questionnaire | Convenience sampling | N.R. | Paper survey | Single | 1 | N/A | N/A | N/A | N.R. | 12 of 38 subjects (31.6%, P = .005) selecting robot-assisted surgery in the marketing frame and not the evidence-based frame |
| Herling et al., Denmark | N.R. (interview style) | Semi-structured in person (clinic) interviews | Criterion-based sampling | Surgeons contacted potential informants by phone | Digitally recorded interviews | Single | 1 | N/A | Mean of 12 weeks post-op | 12 weeks (median) | Thematic analysis | N/A |
| Irani et al., USA | 8 | In person (clinic) self-administered survey questionnaire | Convenience sample | Approached at time of clinic | Paper questionnaire | Single | 1 | N/A | Unclear | No follow up | N/A | (1). Chi-square or Fisher's exact test. (2). Odds Ratio with 95% CI |
| Reynolds et al., Australia | 29 | Self-administered survey questionnaire (postal), and focus group discussions | All eligible participants contacted | N.R. | Paper questionnaire, followed by focus groups in which patient's discussed their experiences | Single | 1 | N/A | Single questionnaire postoperatively (timing unspecified). | N.R. | Thematic analysis for qualitative data | N.R. |
| Chu et al., USA | 29 | In person (clinic) self-administered survey questionnaire | All eligible participants contacted | Approached at time of clinic | Paper questionnaire | Single | 1 | Yes | No | No follow up | N/A | (1). χ2 test or Wilcoxon rank sum test for associated between knowledge nad preference in treatment. (2). Multiple Regression for adjusting associations for possible confounders. |
| Stai et al., USA | 2 | In person (Minnesota State Fair) self-administered survey questionnaire | Convenience sampling | Volunteers compensated with $5 | Digital survey on tablets | Single | 1 | N/A | N/A | No follow up | N/A | Multiple Regression to determine if population characteristics influenced responses |
| Pagani et al., USA | 30 | Online self-administered survey questionnaire | N/A (self-volunteered) | Volunteers compensated with $0.12 | Online questionnaire | Single | 1 | N/A | N/A | No follow up | N/A | (1). Categorical variables - χ2 test or Fisher's exact test. (2). Factors associated independently with preference for RAS - Multivariable logistic regression model |
| Patel et al., Canada | N.R. | Telephone-administered survey questionnaire | All patients | Approached by research team later | Questionnaire asked by interviewers | Single | 1 | No | Yes | No follow up | N/A | Percentages. Statistics used by N.D. |
| Muaddi et al., Canada | N.R. | Online survey questionnaire | Randomized to vignettes comparing laparoscopic surgery to robotic surgery | Participants compensated $1.67 USD per 10 mins | Online survey | Single | 1 | NA | N/A | N/A | N/A | N.R. |
| Claydon et al., UK | N.R. (interview style) | Semi-structured telephone interviews | Rich data inclusion and purposive sampling | Approached by research team pre-operatively | Interview schedule | Single | 1 | No | Yes | N.R. (at least 6 wks) | Braun and Clarke's six-step method of inductive thematic analysis. | N/A |
| Moloney et al., Ireland | N.R. (interview style) | Semi-structured telephone interviews | N.D. | Contacted by surgeon | Interview guide | Single | 1 | No | Yes | No follow up | Thematic analysis | N/A |
| Wu et al., China | N.R. (interview style) | Semi-structured in person (clinic) interviews | Purposive sampling method and maximum variation sampling strategy | Approached by research team later | Face to face interviews | Single | 1 | No | Yes | No follow up | Thematic analysis | N/A |
| Abdelaal et al., USA | 25 | In person (clinic) self-administered survey questionnaire | All eligible participants contacted | Approached at time of clinic | Anonymous paper surveys | Single | 1 | Yes | No | No follow up | N/A | Logistic Regression to determine if population characteristics and surgeon preferences influenced responses |
| Pinci et al., Puerto Rico | 21 | In person (clinic) self-administered survey questionnaire | All patients | Voluntary enrolment, informed consent | Paper questionnaire | Single | 1 | N/A | N/A | No follow up | N/A | (1). Categorical variables - Pearson's χ2 test or Fischer's exact test. (2). Factors associated independently with preference for RAS - Multivariable logistic regression model. OR w/ 95% CI, p-values |
| Chang et al., USA | 16 | In person (clinic) self-administered survey questionnaire | Nonstructured convenience sampling | Distributed by registration staff members | Paper questionnaire | Single | 1 | Yes | No | No follow up | N/A | (1). Descriptive statistics - subgroup analysis of demographics. (2). Spearman's correlation with Rs correlation coefficient, Mann-Whitney U, one-way ANOVA tests with F-value (intergroup variance: intragroup variance) |
| Ashmore et al., UK | N.R. (interview style) | Semi-structured in person (clinic) interviews | Phase 1: Purposeful sampling of patients who had undergone RAS. Phase 2: Block-randomized crossover study, recruiting women >18  from the general public. | N.R. | Phase 1: Semi-structured face-to-face interviews with patients.  Phase 2: Three questionnaires after reviewing RAS information via printed leaflets and a video. | Phase 1 – single.  Phase 2 – 2. | 2 | No | No | No follow up | Thematic analysis for qualitative data | Quantitative data summarised and analysed using Mann-Whitney U and Fishers exact test |
