# Supplementary material for: From expectations to experiences: a systematic review of patient and public perspectives on robotic surgery
Source: J Robot Surg. 2025 Aug 14;19(1):484. doi: 10.1007/s11701-025-02649-y (PMC12354569; doi:10.1007/s11701-025-02649-y)
Supplement: Supplementary file 4 — Supplementary file4 (DOCX 28 KB) [file 11701_2025_2649_MOESM4_ESM.docx]

**Supplementary Information**

**From Expectations to Experiences: A Systematic Review of Patient Perspectives on Robotic Surgery**

*B Jauniaux^1^, A Anand^2^, R Abbas^2^, DP Harji^1,3,4^*

Benoit Jauniaux*,* ^1^Department of Colorectal Surgery, Manchester University NHS Foundation Trust, Manchester, UK*;* [benoit.jaunaux@doctors.org.uk](mailto:benoit.jaunaux@doctors.org.uk), ORCID ID 0000-0002-2527-2112

Ajitesh Anand, ^2^University of Manchester, Manchester, UK; ajitesh.anand@doctors.org.uk, ORCID ID 0000-0003-0184-841X

Rahma Abbass, ^2^University of Manchester, Manchester, UK; [rahmaaabbas02@gmail.com](mailto:rahmaaabbas02@gmail.com), ORCID ID 0009-0002-7966-5277

Deena Harji*,* ^1^Department of Colorectal Surgery, Manchester University NHS Foundation Trust, Manchester, UK, ^4^Robotics and Digital Surgery Initiative, Royal College of Surgeons of England, England, ^5^ Clinical Trials Research Unit, Leeds Institute of Clinical Trials Research, University of Leeds, Leeds, UK; d.harji@leeds.ac.uk ORCID ID 0000-0002-8493-3312

**Corresponding author:** Deena Harji, ^1^Department of Colorectal Surgery, Manchester University NHS Foundation Trust, Manchester, UK, ^4^Robotics and Digital Surgery Initiative, Royal College of Surgeons of England, England, ^5^Clinical Trials Research Unit, Leeds Institute of Clinical Trials Research, University of Leeds, Leeds, UK; d.harji@leeds.ac.uk

**Table S5. A: Quality assessment of all quantitative and mixed-methods studies. B: Quality assessment of qualitative studies.**

| 1. Quality assessment of all quantitative studies | | | | | | | | | | |  |
| --- | --- | --- | --- | --- | --- | --- | --- | --- | --- | --- | --- |
| Author(s), country | | Did the study address a clearly focused issue? | Did the authors use an appropriate method to answer their question? | Were the subjects recruited in an acceptable way? | Were the measures accurately measured to reduce bias? | Were the data collected in a way that addressed the research issue? | Did the study have enough participants to minimise the play of chance? | How are the results presented and what is the main result? | Was the data analysis sufficiently rigorous? | Is there a clear statement of findings? | Can the results be applied to the local population? |
| Ryan et al., USA | * | * |  |  | * |  | * |  | * |  |  |
| El Douaihy et al., USA | * | * |  |  | * |  | * | * | * |  |  |
| Dixon et al., Canada | * | * |  |  | * |  | * |  | * |  |  |
| Irani et al., USA | * | * |  |  | * |  | * |  | * |  |  |
| Reynolds et al., Australia | * | * |  |  | * |  | * |  | * |  |  |
| Chu et al., USA | * | * |  |  | * |  | * |  | * |  |  |
| Stai et al., USA | * | * | * | * | * |  | * | * | * |  |  |
| Pagani et al., USA | * | * | * | * | * |  | * | * | * |  |  |
| Patel et al., Canada | * | * | * |  | * |  | * |  | * |  |  |
| Muaddi et al., Canada | * | * | * |  | * |  | * |  | * |  |  |
| Abdelaal et al., USA | * | * | * |  | * |  | * | * | * |  |  |
| Pinci et al., Puerto Rico | * | * | * | * | * |  | * | * | * |  |  |
| Chang et al., USA | * | * | * | * | * |  | * |  | * |  |  |
| Ashmore et al., UK | * | * |  |  | * |  | * |  | * |  |  |
|  |  |  |  |  |  |  |  |  |  |  |  |
| 1. Quality assessment of qualitative studies | | | | | | | | | | |  |
| Author(s), country | Clear statement of aims | Is qualitative methodology appropriate | Was the research design appropriate to address the aims of the research? | Was the recruitment strategy appropriate to the aims of the research? | Was the data collected in a way that addressed the research issue? | Has the relationship between researcher and participant been adequately considered? | Have ethical issues been taken into consideration? | Was the data analysis sufficiently rigorous? | Is there a clear statement of findings? | How valuable is the research? |  |
| Herling et al., Denmark | * | * | * |  | * | * | * |  | * |  |  |
| Claydon et al., UK | * | * | * | * | * |  | * |  | * | * |  |
| Moloney et al., Ireland | * | * | * | * | * |  | * |  | * | * |  |
| Wu et al., China | * | * | * | * | * |  | * |  | * | * |  |
