# Supplementary material for: From expectations to experiences: a systematic review of patient and public perspectives on robotic surgery
Source: J Robot Surg. 2025 Aug 14;19(1):484. doi: 10.1007/s11701-025-02649-y (PMC12354569; doi:10.1007/s11701-025-02649-y)
Supplement: Supplementary file 5 — Supplementary file5 (DOCX 35 KB) [file 11701_2025_2649_MOESM5_ESM.docx]

**Supplementary Information**

**From Expectations to Experiences: A Systematic Review of Patient Perspectives on Robotic Surgery**

*B Jauniaux^1^, A Anand^2^, R Abbas^2^, DP Harji^1,3,4^*

Benoit Jauniaux*,* ^1^Department of Colorectal Surgery, Manchester University NHS Foundation Trust, Manchester, UK*;* [benoit.jaunaux@doctors.org.uk](mailto:benoit.jaunaux@doctors.org.uk), ORCID ID 0000-0002-2527-2112

Ajitesh Anand, ^2^University of Manchester, Manchester, UK; ajitesh.anand@doctors.org.uk, ORCID ID 0000-0003-0184-841X

Rahma Abbass, ^2^University of Manchester, Manchester, UK; [rahmaaabbas02@gmail.com](mailto:rahmaaabbas02@gmail.com), ORCID ID 0009-0002-7966-5277

Deena Harji*,* ^1^Department of Colorectal Surgery, Manchester University NHS Foundation Trust, Manchester, UK, ^4^Robotics and Digital Surgery Initiative, Royal College of Surgeons of England, England, ^5^ Clinical Trials Research Unit, Leeds Institute of Clinical Trials Research, University of Leeds, Leeds, UK; d.harji@leeds.ac.uk ORCID ID 0000-0002-8493-3312

**Corresponding author:** Deena Harji, ^1^Department of Colorectal Surgery, Manchester University NHS Foundation Trust, Manchester, UK, ^4^Robotics and Digital Surgery Initiative, Royal College of Surgeons of England, England, ^5^Clinical Trials Research Unit, Leeds Institute of Clinical Trials Research, University of Leeds, Leeds, UK; d.harji@leeds.ac.uk

**Table S6. Summary findings of the quantitative studies included.**

| Authors, country | Theme(s) | Questionnaires Used | Specific tool used | Results by Theme | Comments |
| --- | --- | --- | --- | --- | --- |
| Ryan et al., USA | **(1) Factual Knowledge & Understanding** | Study-specific | 10/14 questions dichotomous, 4 open-ended but converted to categorical data | Pre-operative and operative understandings questions answered affirmatively or correctly ranged from 69% to 96%. | Results were irrelevant of education status. Highlights the importance of preoperative education in ensuring patient understanding of robotic-assisted surgery. |
|  | **(2) Experiences & Satisfaction** |  |  | 96% were satisfied with the pre-operative information. 88.8% felt they benefitted from having a robotic approach and would choose robotic surgery again. |  |
| El Douaihy et al., USA | **(1) Perception and Expectations** | Study-specific | Likert scale | 91% of patients perceived robotic-assisted surgery as less invasive compared to traditional open surgery. | A structured preoperative education and counselling program contributed to high satisfaction rates (93%). Continence was the most significant predictor of satisfaction post-surgery (p=0.01) |
|  | **(2) Experiences & Satisfaction** | Study-specific | Likert scale | 92% of patients who received preoperative education about robotic-assisted prostatectomy (RARP), including written materials, video recordings, and explanations from surgeons, felt well-informed about the procedure. 78% of patients felt they had a clear understanding of what the robotic-assisted surgery would entail, compared to only 55% in the group that did not receive structured counselling. |  |
| Dixon et al., Canada | **(1) Preference compared to other methods** | Study-specific | Vignette-associated questions, dichotomous forced-choice format. | Preference was significantly influenced by how RAS was described, indicating susceptibility to language framing (p=0.005). 43.5% of participants preferred RAS when it was described as “innovative” and “state-of-the-art” (marketing group), whereas 25% of the control group preferred RAS. (evidence-based wording) | Marketing language significantly (p<0.001) increases the likelihood that patients will choose robot-assisted surgery, regardless of clinical evidence. Highlights the ethical implications of how RAS is communicated to the public and the importance of neutral, evidence-based patient education. |
| Irani et al., USA | **(1) Preference compared to other methods** | Study-specific | Four-point Likert Scale | 12% open, 33% no pref. No report on how many chose either laparoscopic or robotic. | Correlated with level of education and history of abdominal surgery |
|  | **(2) Factual Knowledge & Understanding** | Study-specific | Three-point Likert Scale | 67.5% did not know how robot works. Higher education (p < 0.05) and history of abdominal surgery (p < 0.05) associated with improved understanding of robot's role | Knowledge level of robotic surgery poor in the cohort. Correlated positively with level of education and history of abdominal surgery. Article suggests those who had to face the reality of having surgery soon likely researched their options more thoroughly, which is probable. |
|  | **(3) Perception and Expectations** | Study-specific | Three-point Likert Scale | 52.6% believed robotic surgery cost more to healthcare than laparoscopic and open surgery. 70% would consult physicians to discuss best options, of which 22.4% would use the internet as first source. | Difficult for clinicians to fathom what patients know about various surgical approaches, increasing complexity of counselling and consent |
|  | **(4) Awareness** | Study-specific | Five-point Likert Scale, Open Answer Question | 46% were not aware of a difference between laparoscopic and robotic modalities. History of abdominal surgery (p < 0.05) associated with improved understanding. |  |
| Chu et al., USA | **(1) Factual Knowledge & Understanding** | Study-specific | Likert scale | Similar knowledge scores between groups (p=0.32). 70% understood that the surgeon performed the actual surgery. | Women presenting with prolapse are unfamiliar with the role, risks, and benefits of robotic-assisted surgery. |
|  | **(2) Preference compared to other methods** | Study-specific | Categorical options | 6.4% preferred robotic approach to pelvic organ prolapse repair. 66% had no preference. 12% preferred laparoscopic. No significance regarding background characteristics. (p = 0.92) |  |
|  | **(3) Perception and Expectations** | Study-specific | Likert scale | Those who preferred robotic felt that it resulted in a shorter operative time than laparoscopic surgery (p<0.001). Most women had neither a positive nor negative impression. |  |
| Stai et al., USA | **(1) Perception and Expectations** | Study-specific | Likert scale | 18% completely uncomfortable with robotic surgery, 37% somewhat uncomfortable, 9% unsure, 26% somewhat comfortable, 10% completely comfortable | Older respondents were more likely to respond "uncomfortable" (p<0.05). Being male ( p = 0.0017), having an associate’s degree (p=0.041), and indicating a zip code with a higher median income ( p = 0.0012) all again had a positive relationship with comfort with robotic surgery. |
|  | **(2) Awareness** | Study-specific | Likert scale | When asked to estimate the percentage of a standard robotic surgery that was autonomous, participants selected values from 0% to 100%. The median value selected was 31.5%, and the mean value was 38.4%. No demographics were found to be significant in multiple regression against this estimation. |  |
| Pagani et al., USA | **(1) Preference compared to other methods** | Study-specific | Three-point Likert Scale | 35% clearly in favour of RAS vs conventional. However, under half would be willing to pay more (41.7%), travel further (43.4%), or wait longer (42.9%) to have RAS. |  |
|  | **(2) Factual Knowledge & Understanding** | Study-specific | Categorical options | 51% of the cohort accurately understood the role of the robot in surgery. 23% believed the robot was completely autonomous. | Significant lack of public awareness regarding robot role in surgery and its associated benefits. Pre-operative education to assist with increased awareness and adoption of robots in surgery. |
|  | **(3) Perception and Expectations** | Study-specific | Categorical options, Binary Likert scale | 59% believed RAS leads to less pain. Public concerns regarding RAS focus on lack of surgeon experience with the robot (28.2%), potential robot malfunction (26.2%), and greater associated cost (22.8%). | Other: Cohort - no confirmation bias (that would otherwise be present in pts planned to undergo robotic Surgery) |
|  | **(4) Awareness** | Study-specific | Three-point Likert Scale, Categorical options, Binary Likert Scale | 16% are very familiar with robotic orthopaedic surgery and a further 59.5% are somewhat familiar. 26.4% know someone who has had robotic orthopaedic surgery before. Most patients who are familiar with robotic surgery have heard of it on TV or seen it on internet. |  |
| Patel et al., Canada | **(1) Experiences & Satisfaction** | Study-specific | Likert scale | 85.89% of the respondents rated their overall experience with robotic surgery as 8+/10. 88.81% were either satisfied or very satisfied with their hospital admission. |  |
|  | **(2) Willingness to pay** | Study-specific | Likert scale | 81.02% expressed a willingness to pay out of pocket for robotic surgery. | Higher income, positive post-operative experiences, and satisfaction with the admission were positively associated with willingness to pay for robotic surgery (p<0.001) |
| Muaddi et al., Canada | **(1) Preference compared to other methods** | Study-specific | Vignette-associated questions | Laparoscopic Surgery was preferred by 64.4% of participants, compared to 35.6% preferring robotic Surgery (p<0.001). | While most participants preferred laparoscopic surgery over robotic surgery (64.4% vs. 35.6%). robotic surgeons were perceived as more competent (55.2%) and trustworthy (53.5%). Findings imply that public preference may be shaped by marketing and perceptions of technology, not actual demand. Fear of robotic surgery didn’t prevent people from associating it with higher skill and technological advancement, highlighting that fear does not equate to preference. |
|  | **(2) Perception and Expectations** | Study-specific | As above | 72.1% of participants believed robotic surgery had a higher chance of surgical error, compared to 18.8% who perceived laparoscopic surgery as having a higher chance of error (p<0.001) 55.2% perceived surgeons performing robotic surgery as more competent, compared to 29.6% who perceived surgeons performing laparoscopic surgery as more competent (p<0.001) | Additionally, 194 (53.5%) have more trust in surgeons who performed robotic surgery, while 130 (36.0%) have more trust in laparoscopic surgeons and 38 (10.5%) expressed no differences, (p < 0.001) |
| Abdelaal et al., USA | **(1) Factual Knowledge & Understanding** | Study-specific | Multiple-choice, qualitative, binary, and Likert scale | Half of the participants expressed uncertainty regarding the function of robots in each step of the Total knee arthroplasty (TKA). | The majority of participants needed more adequate knowledge on robotic surgery. |
|  | **(2) Awareness** | Study-specific | As above | 40% had never even heard about robot assisted technology |  |
|  | **(3) Perception and Expectations** | Study-specific | As above | 49% rated their understanding of robotic surgery as poor or very poor. 55.3% believed hospitals that provide RA-TKA are superior to those that do not. 98% indicated that they want the surgeon to take an active role in making intraoperative decisions. The most frequent concerns were about robot malfunction (55.2%), the surgeons playing a less active part in surgery (48.1%), and the lack of evidence for RA-TKA superiority (28.3%) | Respondents were apprehensive about performance expectations and expressed concerns about robot malfunction during the procedure. This likely stems from misperceptions about the robot's exact role. |
|  | **(4) Preference compared to other methods** | Study-specific | As above | 60% were indecisive or preferred that robotic technology not be used during their TKA surgery. | The collaborative role of the surgeon and robot during the procedure is an area that can be emphasized so patients understand the role of the robot in augmenting the capacity of the surgeon, rather than autonomously performing the surgery. |
|  | **(5) Willingness to pay** | Study-specific | As above | 8.7% would consider paying for the RA-TKA procedure | Patients of RA-TKA advocate surgeons showed a greater willingness to undergo and pay a premium. |
| Pinci et al., Puerto Rico | **(1) Preference compared to other methods** | Modified to Spanish from Pagani et al. | Binary Likert Scale | 53% prefer robotic over manual | Clear bisection in patient preference. Proposed due to personal decision-making influenced by multiple patient-specific factors and circumstances. Patient's needs require better addressing and comprehensive information should be provided. |
|  | **(2) Factual Knowledge & Understanding** | Modified to Spanish from Pagani et al. | Categorical options | 70% of cohort accurately understood the role of the robot in surgery. 10% believed robot was completely autonomous. |  |
|  | **(3) Perception and Expectations** | Modified to Spanish from Pagani et al. | Categorical options, Three-point Likert Scale, Binary Likert Scale | Belief that robotic-assisted surgery leads to better outcomes (54.7%), a faster recovery (53.1%), fewer complications (50%), and more surgical precision (67.8%). | Significant higher costs are associated with robotic total joint arthroplasty (TJA). They must be reduced for widespread implementation. Can have an impact in low-income regions with low case volume. Discrepancies exist between general public's beliefs regarding rTJA and current evidence in literature. |
|  | **(4) Awareness** | Modified to Spanish from Pagani et al. | Three-point Likert Scale, Categorical options, Binary Likert Scale | 56% not familiar, 38% somewhat, 6% very familiar 30% never heard of robot surgery. Out of those who have heard it, most know about it from TV/internet. Only 2.6% have had robotic surgery previously. 15% know of someone who has had robotic surgery. | Survey distribution method can acknowledge differences between this study's cohort's familiarity of rTJA (44.2%) vs Pagani et. Al (70%). Other: Demographic profile similar to national average demographics of Hispanics from US Census Data |
| Chang et al., USA | **(1) Factual Knowledge & Understanding** | Study-specific | Three-point Likert Scale | 30.8% patients believed robots could independently perform 'most' or 'all' of robotic total joint arthroplasty (TJA) operations. |  |
|  | **(2) Perception and Expectations** | Study-specific | Five-point Likert Scale, Categorical options | 77.8% patients were interested in rTJA to 'some degree'. 35% were very interested or extremely interested in RAS.  Interest level positively correlated with age (p = 0.010), education level (p = 0.002), household income (p < 0.001) and White race (p = 0.016). No significant correlation with gender (p = 0.111). 31.9% perceived robots had improved accuracy, 28.3% perceived it provided surgical ease; 30.8% perceived rTJA was more expensive and poses a significant learning curve for surgeons | Education, income and age are correlated with favour for robotic surgery likely due to more understanding and interest. However, correlation with age was weak (therefore minimal significance). Correlation with income and education were sub-moderate |
|  | **(3) Awareness** | Study-specific | Binary Likert Scale | 11.4% had first-degree relative/personal experience with rTJA. Use of robotic surgery in public vs private practice was reported to be about the same by patients (i.e., 30-50% of operations). | Appropriate preoperative counselling, shared decision making and patient selection suggested in conclusion. Research into marketing campaigns as a future direction was unique to this article |
| **Reynolds et al., Australia* | **(1) Experiences & Satisfaction** | Study-specific | Five-point Likert Scale | 97.6% were satisfied/extremely satisfied. 91% would recommend the procedure. 66.9% stated their questions were well answered. | Positive experiences reported more than negative experiences. Findings described generally (lacking specifics). |
|  | **(2) Perception and Expectations** | Study-specific | Five-point Likert Scale | 52.7% were worried/anxious before the surgery |  |
|  | **(3) Awareness** | Study-specific | Five-point Likert Scale | 92.3% prepared in some way for the procedure (e.g., exercises, weight loss, research) |  |
| **Ashmore et al., UK* | **(1) Preference compared to other methods** | Study-specific | Four-point Likert Scale | 43.7% increase (from 31.3 % to 75.0 %) in participants who would choose robotic surgery over laparoscopic or open surgery, after watching the information video. |  |
|  | **(2) Perception and Expectations** | Study-specific | Four-point Likert Scale | The acceptance of RAS improved after receiving informational materials. This was greater in patients receiving pre-operative video information versus leaflet. | Twenty-two (73.3 %) of the participants felt that the video alone was an adequate source of information, compared to only 14 (46.7 %) for the printed leaflet alone. |

*quantitative results from mixed-methods studies.
