# Supplementary material for: From expectations to experiences: a systematic review of patient and public perspectives on robotic surgery
Source: J Robot Surg. 2025 Aug 14;19(1):484. doi: 10.1007/s11701-025-02649-y (PMC12354569; doi:10.1007/s11701-025-02649-y)
Supplement: Supplementary file 6 — Supplementary file6 (DOCX 24 KB) [file 11701_2025_2649_MOESM6_ESM.docx]

**Supplementary Information**

**From Expectations to Experiences: A Systematic Review of Patient Perspectives on Robotic Surgery**

*B Jauniaux^1^, A Anand^2^, R Abbas^2^, DP Harji^1,3,4^*

Benoit Jauniaux*,* ^1^Department of Colorectal Surgery, Manchester University NHS Foundation Trust, Manchester, UK*;* [benoit.jaunaux@doctors.org.uk](mailto:benoit.jaunaux@doctors.org.uk), ORCID ID 0000-0002-2527-2112

Ajitesh Anand, ^2^University of Manchester, Manchester, UK; ajitesh.anand@doctors.org.uk, ORCID ID 0000-0003-0184-841X

Rahma Abbass, ^2^University of Manchester, Manchester, UK; [rahmaaabbas02@gmail.com](mailto:rahmaaabbas02@gmail.com), ORCID ID 0009-0002-7966-5277

Deena Harji*,* ^1^Department of Colorectal Surgery, Manchester University NHS Foundation Trust, Manchester, UK, ^4^Robotics and Digital Surgery Initiative, Royal College of Surgeons of England, England, ^5^ Clinical Trials Research Unit, Leeds Institute of Clinical Trials Research, University of Leeds, Leeds, UK; d.harji@leeds.ac.uk ORCID ID 0000-0002-8493-3312

**Corresponding author:** Deena Harji, ^1^Department of Colorectal Surgery, Manchester University NHS Foundation Trust, Manchester, UK, ^4^Robotics and Digital Surgery Initiative, Royal College of Surgeons of England, England, ^5^Clinical Trials Research Unit, Leeds Institute of Clinical Trials Research, University of Leeds, Leeds, UK; d.harji@leeds.ac.uk

**Table S7. Synthesised themes and categories in qualitative and mixed-methods studies.**

| **Theme(s)** | **Category** |
| --- | --- |
| Factual Knowledge & Understanding | Robot's operator |
|  | Robot function |
|  | Robot set-up |
|  | Post-op recovery |
|  | Use of video tools |
| Awareness | Pre-op information sourcing |
|  | Wider aspects of the procedure |
|  | Risks and side effects |
|  | Cost |
|  | Acceptance of information |
| Perception and Expectations | Robot's role |
|  | Robot's precision |
|  | Robot's appearance |
|  | Trust in surgical team |
|  | Trust in robot |
|  | Robot malfunction |
|  | Post-op recovery |
|  | Potential psychological impact |
| Preference compared to other methods | Autonomy of choice |
|  | Informed decision-making |
|  | General advantages and disadvantages |
|  | Post-operative recovery impacts |
|  | Invasiveness |
|  | Urgency |
| Experiences & Satisfaction | Lack of information |
|  | Method of information provision |
|  | Physical health impacts |
|  | Psychological impact |
|  | Post-op recovery |
|  | Wider care provision |
| Willingness to pay | Cost |
